# Addiction Symptom Network of Young Internet Users: Network Analysis

**DOI:** 10.2196/38984

**Published:** 2022-11-10

**Authors:** Jianxia Lu, Qinhan Zhang, Na Zhong, Jin Chen, Yujia Zhai, Lei Guo, Chunlei Lu, Tianzhen Chen, Zhongli Jiang, Hui Zheng

**Affiliations:** 1 School of Rehabilitation Jiangsu Vocational College of Medicine Yancheng China; 2 College of Teacher Education Zhejiang Normal University Jinhua China; 3 Shanghai Key Laboratory of Psychotic Disorders Shanghai Mental Health Center Shanghai Jiao Tong University School of Medicine Shanghai China; 4 Department of Rehabilitation Medicine The First Affiliated Hospital of Nanjing Medical University Nanjing China

**Keywords:** internet addiction, Internet Addiction Test, network analysis, adolescents

## Abstract

**Background:**

An increasing number of people are becoming addicted to the internet as a result of overuse. The Internet Addiction Test (IAT) is a popular tool for evaluating internet use behaviors. The interaction between different symptoms and the relationship between IAT and clinical diagnostic criteria are not well understood.

**Objective:**

This study aimed to explore the core symptoms of internet addiction (IA) and the correlation between different symptoms of the IA symptom network. Network analysis was also conducted to explore the association between the IAT scale and the Diagnostic and Statistical Manual of Mental Disorders–5th edition (DSM-5) criteria for IA.

**Methods:**

We recruited 4480 internet users (aged 14-24 years), and they completed the IAT. The final analysis included 63.50% (2845/4480) of the participants after screening the submitted questionnaires. Participants were classified into IA group and non-IA (NIA) group. By using partial correlation with Lasso regularization networks, we identified the core symptoms of IA in each group and compared the group differences in network properties (strength, closeness, and betweenness). Then, we analyzed the symptom networks of the DSM-5 diagnostic criteria and IAT scale for IA.

**Results:**

A total of 12.47% (355/2845) of the patients were in the IA group and 87.52% (2490/2845) of the patients were in the NIA group, and both groups were evaluated for the following nodes: IAT_06 (school work suffers; strength=0.511), IAT_08 (job performance suffers; strength=0.531), IAT_15 (fantasize about being on the web; strength=0.474), IAT_17 (fail to stop being on the web; strength=0.526), and IAT_12 (fear about boredom if offline; strength=0.502). The IA groups had a stronger edge between IAT_09 (defensive or secretive about being on the web) and IAT_18 (hidden web time) than the NIA groups. The items in DSM-5 had a strong association with IAT_12 (weight=−0.066), IAT_15 (weight=−0.081), IAT_17 (weight=−0.106), IAT_09 (weight=−0.198), and IAT_18 (weight=−0.052).

**Conclusions:**

The internet use symptom network of the IA group is significantly different from that of the NIA group. Nodes IAT_06 (school work affected) and IAT_08 (work performance affected) are the resulting symptoms affected by other symptoms, whereas nodes IAT_12 (fear about boredom if offline), IAT_17 (inability to stop being on the web), and IAT_15 (fantasize about being on the web) are key symptoms that activate other symptoms of IA and are strongly linked to the inability to control the intention to play games in the DSM-5.

## Introduction

### Background

Internet addiction (IA) refers to the inability of individuals to control their internet behaviors, which can also lead to dysfunction in their lives [1]. The prevalence of IA varies from 0.8% to 26.7% in different populations, with high prevalence in adolescents and young adults [2]. IA leads to serious dysfunction symptoms, which are often characterized by irritability, quarrels with people, increased lying, academic neglect, social withdrawal, and mental symptoms [3]. Currently, there is no unified standard for the diagnosis of IA. The recently proposed Assessment of Criteria for Specific Internet-use Disorders mainly refers to International Classification of Diseases 11th revision (ICD-11) [4]. The treatment of IA remains in the mapping stage [5]. However, the diagnosis and treatment of IA are complicated by the lack of clarity regarding the core symptoms and their interactions.

Addiction is generally considered to have symptoms such as abnormal craving desires, impaired inhibitory control, compulsive addictive behaviors, and abnormalities in negative emotion regulation [6]. By comparing ICD-11 and Diagnostic and Statistical Manual of Mental Disorders–5th edition (DSM-5) diagnostic criteria, we found 2 characteristics mentioned by both. First, people not only spend a lot of time and energy in playing games but, more importantly, they also ignore the realities of life and can no longer undertake the social roles they used to play and no longer participate in social life. Second, they lose control of their actions and let the games control their lives. The reason for both is that individuals cannot be satisfied in the real world but show their spiritual world on the internet to obtain a sense of achievement [7]. Such a perfect image, which is different from that of the real world, is brought about by the anonymity of the internet. Anonymity may amplify negative symptoms, thereby influencing other symptoms of IA [8]. The core symptoms are the most influential symptoms of a disorder, and they can activate other symptoms and promote the development of mental illness [9]. In general, identifying the core symptoms helps to target clinical interventions. Core symptoms are classified as key symptoms and outcome symptoms based on their different properties. Key symptoms are the core symptoms that have the greatest impact on other symptoms in the network, and outcome symptoms are the most affected by other symptoms [10].

In recent years, the emerging perspective of network analysis has provided new view and tools for understanding psychopathology [11]. In network analysis, which is different from the previous perspective, the existing models conceptualize psychiatric disease as a static structure based on underlying models, suggesting that observable clinical symptoms and signs are caused by underlying variables [12-16]. The symptom network perspective considers symptoms as the integrated component of mental disorders [9,17]. The network analysis approach can identify the most influential symptoms (core symptoms) in the symptom network, which are defined as the symptoms with high centrality. These influential symptoms can more actively affect other symptoms in the network, thus promoting the development of mental illness [9]; therefore, identifying the influential symptoms is helpful in identifying targets for clinical intervention. *Strength* and *edge* are two important indices in network analysis, and *strength* represents the total weight of the connections from other nodes to specific nodes. The association line (or *edge*) between the node is represented. *Edge* is a line between nodes that represents a regularization part. The wider the edge, the stronger the association.

Clinical symptoms of IA are often measured using questionnaires such as the Internet Addiction Test (IAT) [18,19]. The IAT is a widely used tool, and it shows adequate psychometric features [20]. There is evidence that the IAT is a valid and reliable scale for the screening of IA among Chinese adolescents [21]. The IAT scale contains a total of 20 items and can reflect IA from compulsive use behaviors, withdrawal symptoms, tolerance, interpersonal health, time management, and other aspects [21]. Although the total score of multiple items can reflect the degree of the disease, there is still no conclusion on how to treat the symptoms; one of the reasons is that the relationship between symptoms is not clear. IA affects adolescents’ daily life; for instance, learning is an important part of life and is reflected in two items: IAT_06 (school work suffers) and IAT_08 (job performance suffers). In addition, IAT_09 (defensive or secretive about being on the web) and IAT_18 (hidden web time) involve the concealment of information related to internet access, which is related to internet anonymity; therefore, there may be a certain connection between them. Previous studies have explored the relationships between different symptoms from several perspectives [22], but there are only few conclusions [13]. A small sample of investigations on autism have found defensive and secretive behaviors and the concealment of internet use to be core symptoms [13], whereas a small sample of studies on depression have found that the most important bridge symptom is node IAT_11 (“Anticipation for future online activities”), followed by IAT_12 (“Fear that life is boring and empty without the internet”) and IAT_19 (“Spend more time online over going out with others”) [23]. However, studies of large samples of IA groups are still lacking.

### Objectives

Therefore, the purpose of this study was to explore the core symptoms of IA using network analysis and the associations between different symptoms in the IA symptom network. We additionally included symptom measure entries from the DSM-5, similar to other 9-item Internet Gaming Disorder Scale–Short Form (IGDS9-SF) questionnaires [24]. On the basis of previous studies, we propose 2 hypotheses. Hypothesis 1 is that the symptom network is different between IA and non-IA (NIA) groups, and it is determined by comparing ICD-11 and DSM-5. Hypothesis 2 is that craving for the internet, loss of control, and negative emotional state are the main symptoms of IA [7].

## Methods

### Participants

Approximately 5900 college students—all with internet experience—from 2 universities in Jiangsu, China, were invited via advertisement to participate in this study. This study was a single-center sampling study. In September 2019, 15.18% (896/5900) of the participants completed the questionnaires. Then, in May 2020, 60.74% (3584/5900) of the participants completed the questionnaires. After we sifted through the 2 batches of questionnaires submitted, 36.49% (1635/4480) of the participants were excluded because they chose the same answer for ≥7 consecutive questions in the questionnaire. This was most likely because they did not fill in the questionnaire carefully, which will lead to inaccurate completion of the questionnaire and affect the accuracy of the final data analysis results. Therefore, 63.50% (2845/4480) of the participants were included in the final analysis. The first group of included participants reported their internet use history and IAT scale on a reliable, web-based data-collection survey platform in China [25]. The second batch had 9 DSM-5 questions about IA in addition to the original questionnaire. All available students (4480/4480, 100%) were fully informed of the purpose of the investigation and participated voluntarily.

### Ethics Approval

This study was approved by the research ethics committee of Jiangsu Vocational College of Medicine (2020002). All participants in the survey and their parents and schools signed the informed consent forms.

### Measures

The participants’ demographic information, including gender, age, and internet use history (participants’ common internet access methods and activities and internet access frequency) were collected using a homemade structured survey.

The IA severity of the participants was assessed using a self-evaluated instrument: the IAT scale [26]. The score for each item ranges from 1 (rarely) to 5 (always). The total score of the IAT scale ranges from 20 to 100, and a high score is indicative of severe IA. The IAT is a valid and reliable scale with satisfactory internal consistency (Cronbach *α*=.84). According to a previous study, IAT score ≥50 is indicative of IA [13]. Those with IAT scale score <50 were allocated into the NIA group. The abbreviations for the IAT scale are shown in Table 1.

The criteria for DSM-5 were revised with reference to the IGDS9-SF (which contains all 9 Internet Gaming Disorder criteria proposed by the American Psychological Association in the DSM-5) and were used to aid in the diagnosis of IA, along with the IAT score. The 9 questions used in this study were modified with reference to the IGDS9-SF, under the guidance of clinicians. Internal consistency in the present sample was good (Cronbach *α*=.74) [27]. IA diagnosis depends on nine diagnostic criteria, of which at least five must be met to be diagnosed with IA: (1) preoccupation with internet games, (2) withdrawal symptoms when not playing, (3) tolerance, (4) unsuccessful attempts to reduce or stop playing, (5) gives up other activities to play, (6) continues playing despite problems caused by it, (7) deceives or covers up playing, (8) plays to escape adverse moods, and (9) risks or losses in relationships or career opportunities because of excessive playing.

**Table 1:** Node abbreviations for IAT^a^ scale.

Node	Item	Abbreviation
IAT_01	How often do you find that you stay online longer than you intended?	Stay on the web beyond schedule
IAT_02	How often do you neglect household chores to spend more time online?	Neglect household chores
IAT_03	How often do you prefer the excitement of the internet to intimacy with your partner?	Internet trumps intimacy
IAT_04	How often do you form new relationships with fellow online users?	Make new connections via the web
IAT_05	How often do others in your life complain to you about the amount of time you spend online?	Complained about being on the web for very long
IAT_06	How often do your grades or school work suffer because of the amount of time you spend online?	School work suffers
IAT_07	How often do you check your email before something else that you need to do?	Check email first
IAT_08	How often does your job performance or productivity suffer because of the internet?	Job performance suffers
IAT_09	How often do you become defensive or secretive when anyone asks you what you do online?	Defensive or secretive about being on the web
IAT_10	How often do you block out disturbing thoughts about your life with soothing thoughts of the internet?	Use the web to escape from emotion
IAT_11	How often do you find yourself anticipating when you will go online again?	Craving for next internet use
IAT_12	How often do you fear that life without the internet would be boring, empty, and joyless?	Fear about boredom if offline
IAT_13	How often do you snap, yell, or act annoyed if someone bothers you while you are online?	Annoyed at being interrupted
IAT_14	How often do you lose sleep due to being online?	Lose sleep
IAT_15	How often do you feel preoccupied with the internet when offline, or fantasize about being online?	Fantasize about being on the web
IAT_16	How often do you find yourself saying “just a few more minutes” when online?	Reluctant to be offline
IAT_17	How often do you try to cut down the amount of time you spend online and fail?	Fail to stop being on the web
IAT_18	How often do you try to hide how long you have been online?	Hidden web time
IAT_19	How often do you choose to spend more time online over going out with others?	Prefer using the web than going out
IAT_20	How often do you feel depressed, moody, or nervous when you are offline, which goes away once you are back online?	Web makes you feel better

^a^IAT: Internet Addiction Test.

### Network Creation

The IAT symptom network model was constructed using all 2845 participants and analyzed with R software (version 3.6.1; R Foundation for Statistical Computing) using the *qgraph* package. In this study, the partial correlation network method was used to estimate all symptom networks, including the IAT network and DSM-5 internet-based symptom network. The edge of the network can be understood as partly related quantities. The estimation steps of each part of the related networks are as follows: to estimate the symptom network illustrating the relationship between IA and sleep disturbance symptoms, partial correlation-based sparse Gaussian graphical models with Lasso regularization were constructed. In this procedure, all edges in the network and sets of small edges were shrunk exactly to 0 (the Lasso regularization) [13]. This process of regularization was coupled with best-fit model selection by following an extended Bayesian information criterion, leading to a sparse network with explanatory power [13]. In the network, a circle represents an individual symptom (1 item from IAT or DSM-5). The association line (or *edge*) between the nodes is represented. An *edge* is a line between nodes that represents a regularization part. The existence of the edge indicates the dependence between the nodes of the IAT network. There is no indication of variables that cannot be independent (all other nodes in a given network). The wider the edge, the stronger the association.

To explore the network correlation between the IAT scale and participants’ DSM-5 items, we constructed the internet-based symptom network of these 2 scales using the same methods. For this internet-based symptom network, we further selected data from the second batch of 79.38% (2845/3584) participants.

### Network Properties

We used the three common centrality measures of *strength*, *closeness*, and *betweenness* to quantify the features of the nodes in the IAT symptom network and IAT and DSM-5 internet-based symptom network [28]. *Strength* represents the total weight of the connections from other nodes to specific nodes. For example, in the Netherlands, a prospective longitudinal study of healthy adults, Boschloo et al [29] found that high-intensity minds lacking depressive symptoms (fatigue, depression, pleasure, and attention concentration) reached threshold levels of the crowd; in the next 6 years, there was great risk of severe depressive episodes. *Closeness* is defined as the inverse of the sum of the shortest distances from a particular node to all other nodes in the network. The shortest distance is the minimum number of edges from one node to the next node. High internet closure indicates that the average distance between the given node and all other nodes in the network is short. In epidemiology, patients with high closeness are more likely to trigger the rapid development of an epidemic. *Betweenness* is the number of times the shortest path between any 2 symptoms passes through another symptom. Nodes with high betweenness can be considered as *bridges* to other symptoms; that is, if you remove a high node, the distance between the other nodes generally increases. *Expected influence* is a measurement of centrality that quantifies how strongly and directly a symptom node is associated with all the other nodes in the network. The results from a longitudinal study of older bereaved adults showed that decreased symptom severity with high expected influence predicted clinical improvements across the network compared with complex grief symptoms with low expected influence. Centrality is measured as the shortest path length of any 2 symptoms, and a highly mediated symptom can be considered as a bridge that connects other symptoms.

For network symptoms, nodes with symptoms with high strength can be considered as core symptoms because symptoms of nodes with high strength are more closely related to other symptoms. According to the psychopathological network theory, when a high-strength node is activated, the probability of its symptoms being further activated is relatively high. Symptoms with low node strength can be considered as marginal [28].

### Network Stability Estimates

The accuracy of the edge and stability estimation of the network were calculated using a bootstrapping process of 1000 iterations. First, we estimated the accuracy of the edge of the 95% CI by bootstrapping the edge weight. The overlap between these CIs indicates less accuracy. Second, we tested the stability of the *node* center through a subset of the bootstrapping process. We estimated the central stability (CS) coefficient (CS factor) as a reference indicator. A CS coefficient weight that equals 0.25 indicates acceptable stability.

### Network Comparison

We compared the IAT symptom networks of IA and NIA samples using the Network Comparison Test package in R to explore possible differences in the overall connectivity between IA and NIA. The comparison was based on a permutation procedure, and the number of permutations was 5000.

## Results

### Participant Characteristics

We included 63.50% (2845/4480) of the participants after screening, with 12.47% (355/2845) of the patients in the IA group (mean score 57.32, SD 7.70; mean age 19.36, SD 1.07 years; 89/355, 25.1% men) and 87.52% (2490/2845) of the patients in the NIA group (mean score 34.75, SD 7.87; mean age 18.59, SD 1.69 years; 938/2490, 37.67% men). The present sample was aged 14 to 24 years, and most of them (1203/2845, 42.28%) were aged 18 or 19 years. There were significant differences in age and IAT scores between the IA and NIA groups. There were also significant gender differences in IAT scores.

A descriptive analysis of the internet use habits of 69.49% (1977/2845) of the participants after the second screening (Table 2) indicated that mobile phones were the most dominant way of accessing the internet. Most participants (882/1977, 44.61%) spent an average of 3 to 6 hours a day on the web in the past month.

**Table 2:** Sample characteristics and internet use habits (n=1977^a^).

		IA^b^ (n=344, 17.40%)	NIA^c^ (n=1633, 82.59%)
Age in years, mean (SD)		19.6 (0.852)	19.7 (0.938)
**Gender, n (%)**			
	Men	113 (32.8)	392 (24)
	Women	231 (67.2)	1241 (75.99)
**Main internet access modes, n (%)**			
	Mobile phone	338 (98.3)	1598 (97.85)
	Computer	4 (1.2)	28 (1.71)
	Tablet computer	2 (0.6)	5 (0.31)
	Other^d^	0 (0)	2 (0.12)
**Time spent on the web every day in the last month (hours), n (%)**			
	0-3	36 (10.5)	359 (21.98)
	3-6	130 (37.8)	752 (46.05)
	6-9	91 (26.5)	359 (21.98)
	9-12	43 (12.5)	77 (4.72)
	>12	44 (12.8)	86 (5.27)

^a^Of a total of 2845 participants included, 1977 (69.49%) completed self-reports of internet use.

^b^IA: internet addiction.

^c^NIA: non–internet addiction.

^d^In addition to the 3 modes mentioned, the participants’ main mode of access to the internet.

### Symptom Networks of IA

**Figure 1:**
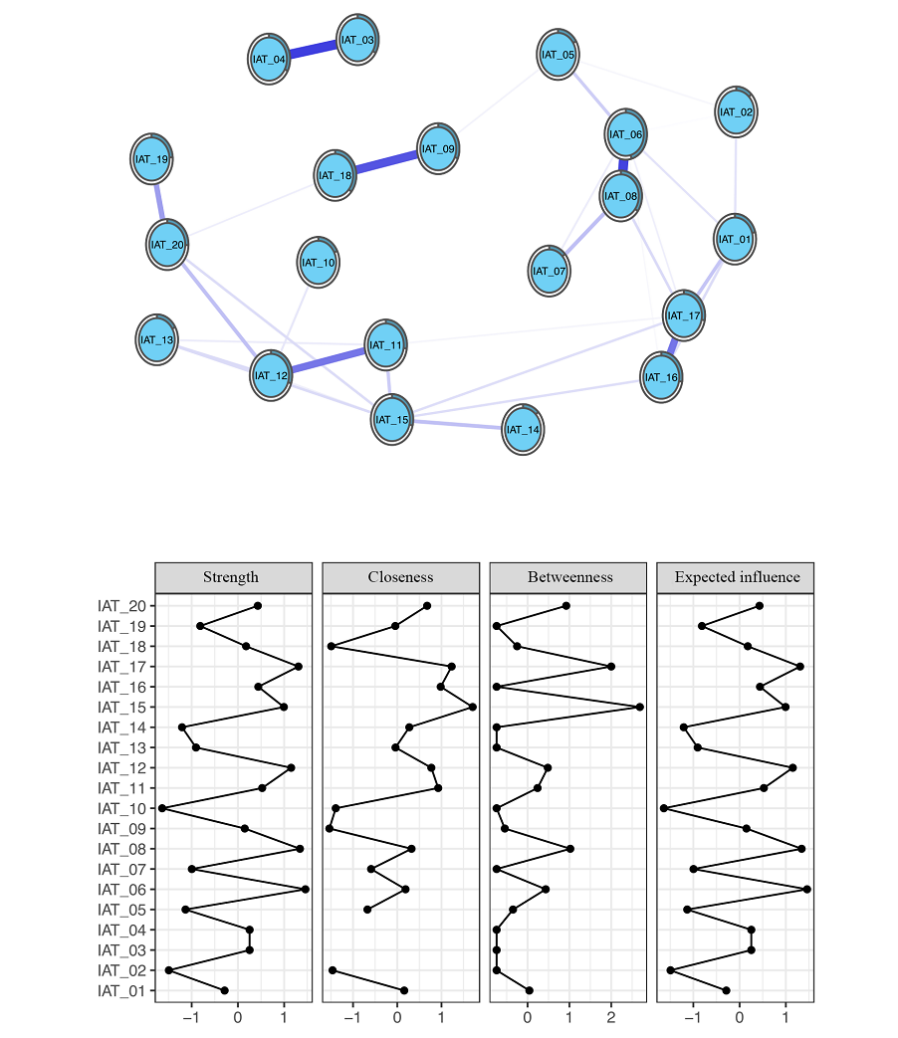
Internet addiction symptom network and centrality measures of the internet addiction group. IAT: Internet Addiction Test.

In Figure 1, the network diagram shows the conditional associations among the 20 questions in the IAT scale for the IA group. The network diagram shows the correlation (circles) and predictability estimates (outer circles) for the 20 items in the IAT scale in the IA group. Each circle in the figure represents an item of the named nodes in the network. The edges represent the strengths of the associations between the nodes. The outer circles represent the level of predictability. The meaning of the title represented by each node abbreviation is provided in Table 1. The most predictable node was IAT_06. Nodes IAT_06 and IAT_08 had the strongest connections.

Centrality measures for the IA symptom network represent the strength, closeness, betweenness, and expected influence value of each node. Higher values indicate that the item is more central in the network. Nodes IAT_06, IAT_08, IAT_17, IAT_12, and IAT_15 were the highest in the strength centrality indices. This indicates that the total weights of the connections from other nodes to nodes IAT_06, IAT_08, IAT_17, IAT_12, and IAT_15 were the highest. Node IAT_15 had the highest closeness, indicating that this cognitive symptom was influential in connecting other symptoms that were otherwise unrelated in the network. Nodes IAT_15 and IAT_17 were the highest in the betweenness centrality indices. This indicates that these 2 highly mediating nodes can be regarded as a bridge to connect other symptoms. Nodes IAT_06, IAT_08, IAT_17, IAT_12, and IAT_15 were the highest in expected influence, indicating that they had the strongest positive associations with other nodes; however, not all other centrality indices were the highest. The meaning of the title represented by each node abbreviation is provided in Table 1.

**Figure 2:**
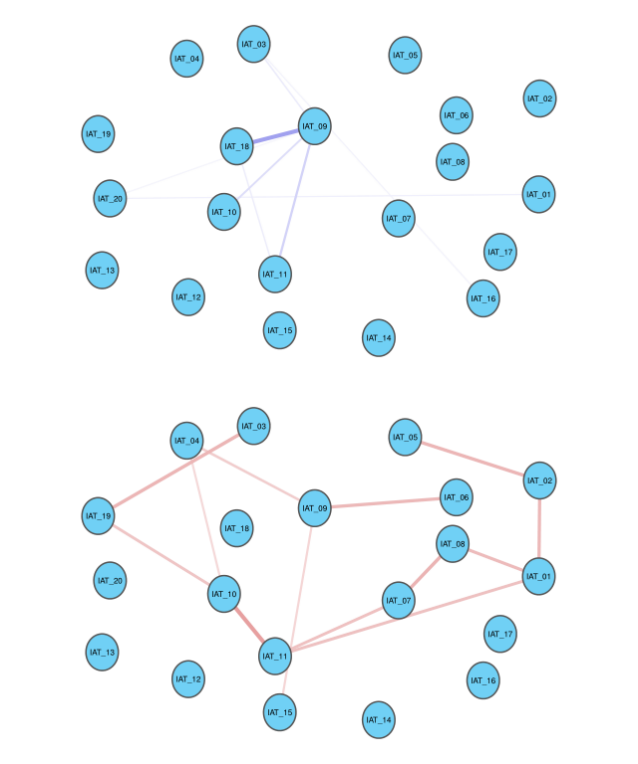
Edges exhibiting significant differences between internet addiction and non–internet addiction groups. IAT: Internet Addiction Test.

Figure 1 shows the constructed symptom network of the IAT scale for the addiction group. As illustrated in Figure 2, IAT_06 (school work suffers; strength=0.511; betweenness=48) and IAT_08 (job performance suffers; strength=0.531; betweenness=72) had high strength.

Moreover, IAT_12 (fear about boredom if offline; strength=0.500; betweenness=50), IAT_17 (fail to stop being on the web; strength=0.526; betweenness=112), and IAT_15 (fantasize about being on the web; strength=0.474; betweenness=140) also had high strength on the IAT scale. These 5 nodes were the 5 points with the highest strength. It is worth noting that these nodes had the highest value on the *strength* measures. These nodes were also significantly higher than those for all other symptoms when comparing the expected influence values.

Furthermore, we found that the values of the *closeness* of IAT_03 (internet trumps intimacy) and IAT_04 (make new connections via the web) were not shown. *Closeness* is defined as the inverse of the sum of the shortest distances from a particular node to all other nodes in the network. According to Figure 1, there is no connection with the other nodes, except for the connection between these 2 nodes; therefore, the total value of both nodes is very extreme and not shown in the figure.

### Comparison of Networks Between the IA and NIA Groups

In Figure 2, the blue edges denote the increased correlations between items in IA compared with those in the NIA group, and the red edges denote the decreased correlations. Edges exhibit significant differences between the IA group and NIA group. The correlation between IAT_18 and IAT_09 in the IA group was significantly stronger than that in the NIA group. The correlation between IAT_10 and IAT_11 in the IA group was significantly weaker than that in the NIA group. The meaning of the title represented by each node abbreviation is provided in Table 1.

We used a permutation-based method to compare edges exhibiting significant differences between individuals with IA and those without IA (NIA); significant positive and negative correlations are shown in Figure 2. Compared with individuals without IA (NIA), IAT_09 (defensive or secretive about being on the web) gained strong connections with IAT_18 (hidden web time; mean difference=0.172; *P*=.01).

In contrast, the symptoms of IAT_11 (craving for next internet use) showed decreased connection with IAT_10 (being on the web to escape from emotion; mean difference=−0.174; *P*=.003). Similarly, IAT_09 (defensive or secretive about being on the web) had weak connection with IAT_15 (fantasize about being on the web; mean difference=−0.073; *P*=.03).

### Symptom Networks of IA and DSM-5

**Figure 3:**
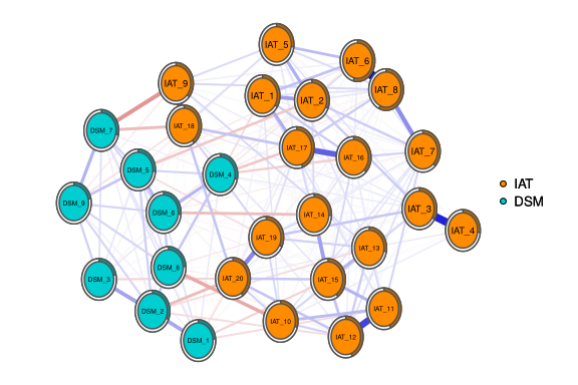
Internet addiction test (IAT) and Diagnostic and Statistical Manual of Mental Disorders–5th edition (DSM-5) symptom network.

In Figure 3, the network graph shows associations and predictability estimates of 20 items in IAT and 9 items in the DSM-5 diagnostic criteria for IA. Each circle in the diagram represents a term of the named node in the network. The outer circle represents the predictable level. The most predictable node was IAT_08. The edges represent the strengths of the associations between the nodes. Nodes IAT_06 and IAT_08 had the strongest connections. The meaning of the title represented by each node abbreviation is provided in Table 1.

The estimated network included 29 nodes, 20 items in IAT, and 9 items in the DSM-5 diagnostic criteria for IA. The estimated network yielded 406 edges (29×[29–1]/2), among which 199 edges had non-0 weights. The weight of the edge connecting IAT_06 (school work suffers) and IAT_08 (job performance suffers) was the strongest (0.479). Other strong associations included those between IAT_03 (internet trumps intimacy) and IAT_04 (make new connections via the web; 0.414) and between IAT_11 (craving for next internet use) and IAT_12 (fear about boredom if offline; 0.353).

Figure 3 shows that node DSM_1 in the DSM-5 diagnostic criteria for the IA community had the strongest weight with node IAT_15 (fantasize about being on the web) in the IAT community (−0.081). Node DSM_2 had a strong weight with node IAT_20 (being on the web makes you feel better; −0.125) and node IAT_12 (fear about boredom if offline; −0.066). Node DSM_4 had the strongest weight with node IAT_17 (failing to stop being on the web; −0.106). Node DSM_7 had the strongest weight with node IAT_09 (defensive or secretive about being on the web; −0.198). Node DSM_9 had the strongest weight with node IAT_18 (hidden web time; −0.052).

## Discussion

### Principal Findings

In this study, the core symptoms of IA were identified by conducting a descriptive analysis of the network symptoms of the IAT scale in the IA and NIA groups. In addition, the difference between the 2 networks was determined by network comparison. From this, we found 2 main results. First, the core symptoms of the IA group were IAT_06 (school work suffers), IAT_08 (job performance suffers), IAT_12 (fear about boredom if offline), IAT_17 (fail to stop being on the web), and IAT_15 (fantasize about being on the web). Second, there were differences in the network between the IA group and NIA group. Together, these results partially support our hypotheses. The results support hypothesis 1 and partially support hypothesis 2. In hypothesis 2, craving the internet and loss of control are the core symptoms of IA, but the results show that a negative emotional state is not the core symptom of IA. Interestingly, the result showed that IAT_09 (defensive or secretive about being on the web) had a strong connection with IAT_18 (hidden web time). This result was also supported by the network connection with DSM-5 symptoms; for example, anonymity was associated with community. Thus, the anonymity of the internet is a potential factor affecting the behavioral performance of IA. The DSM-5 diagnostic criteria for IA have high correlation with key symptoms and low correlation with outcome symptoms.

Craving, losing control, and boredom appeared to play a key role in the development of IA. Our study found that IAT_06 (school work suffers), IAT_08 (job performance suffers), IAT_12 (fear about boredom if offline), IAT_17 (fail to stop being on the web), and IAT_15 (fantasize about being on the web) were the core symptoms of IA, which was consistent with the results of previous studies that found that two items—“Life is boring and empty without the internet” and “Anticipation for future online activities”—were central to the IA network in both groups [13]. Studying is an important part of college students’ lives. Excessive use of the internet has an impact on study and life. This study showed that IAT_06 (school work suffers) and IAT_08 (job performance suffers) nodes were closely related to other symptoms, and other symptoms (eg, IAT_17—fail to stop being on the web) lead to IAT_06 and IAT_08, which affect daily learning and life. Therefore, the core symptoms, IAT_06 and IAT_08, were the most affected by other symptoms.

Interestingly, the other nodes IAT_12 (fear about boredom if offline), IAT_17 (fail to stop being on the web), and IAT_15 (fantasize about being on the web) were key symptoms that activated other IA symptoms. Previous studies have found that individuals with IA have low self-esteem, high feelings of loneliness, and poor social skills [30]. The anonymity of the internet makes it easy to socialize. Individuals with IA are more eager to obtain social satisfaction in the web-based world because their social needs cannot be met in real life. This also causes individuals with IA to rely on the internet and ignore the real world, which has a certain impact on people’s daily lives [31]. After leaving the internet, their sense of estrangement in the real world makes people miss the internet more [32], they cannot control their web-based behavior, and they are dominated and occupied by games. Therefore, for individuals with IA, more attention should be given to building harmonious social circles for them, teaching them certain social skills, and enhancing their self-esteem. In addition, cognitive behavioral therapy may be beneficial because the fear that life without web-based activity will become boring and empty is a core belief of individuals who have difficulty in controlling their internet use. Previous studies have reported the efficacy of cognitive behavioral therapy in reducing IA, reducing internet use, and improving time management skills and emotional stability [13].

Furthermore, connection analysis showed that IAT_09 (defensive or secretive about being on the web) gained strong connections with IAT_18 (hidden web time) when comparing individuals with IA and those without IA (NIA). Both nodes involve the hiding of information related to internet access and anonymity of the internet. Studies have shown that the special nature of web-based communication (anonymity, lack of visual indicators for social discomfort, lack of physical presence, etc) can promote people’s self-disclosure [33,34]. In a web-based environment, where there is no need to be seen, people can change their identities on the web and act as if they are someone else [35]. Therefore, they do not want to let the people around them know, and they want to hide information about the network. The anonymity of the internet is a potential factor affecting the behavioral performance of individuals with IA. This may be related to adolescents’ desire to avoid discipline and stigma. It also suggests that we need to pay attention to the reasons adolescents mask their IA time, especially because Chinese adolescents are generally under academic pressure, playing games is seen as bad behavior, and excessive game use may also make them feel shame and fear about being blamed. Parents and teachers should pay more attention to the balanced development of adolescents and establish good communication relationships with them so that mental health problems can be identified earlier. In addition, for adolescents who are at high risk for addiction, diagnostic assessments should focus on reliable information provided by those around them, rather than on just the individual’s representations, so that people addicted to the internet can be identified more accurately and early [36]. The symptom network of IA and DSM-5 shows that the DSM-5 diagnostic criteria for IA are associated with key symptoms (IAT_12, IAT_15, and IAT_17) and have low correlation with outcome symptoms (IAT_06 and IAT_08). Our study results have found that the symptom networks of IA and DSM-5 are stable. Every criterion of the DSM-5 has a connection with a node of the IAT. On the basis of the IAT scale, the analysis of the core symptoms of IA shows that the diagnostic criteria of DSM-5 have a strong connection with IAT_09 (defensive or secretive about being on the web), IAT_12 (fear about boredom if offline), IAT_15 (fantasize about being on the web), IAT_17 (fail to stop being on the web), and IAT_18 (hidden web time), whereas the connections with IAT_06 (school work suffers) and IAT_08 (job performance suffers) are weak. IAT_06 and IAT_08 mainly emphasize the influence of IA on daily life and learning, whereas the diagnostic criteria of IA in DSM-5 may not lead to judgments of the direct influence of IA on daily learning and life. In addition, the concealment of addictive behavior is a common manifestation of the social stigma associated with addiction in general [37], and in adolescents, this concern can exacerbate other neuropsychiatric symptoms [38]. These findings may provide a practical basis for the diagnostic criteria of DSM-5 IA.

### Limitations and Strengths

This study has several limitations. First, the sample size of the IA group was significantly smaller than that of the NIA group. Second, this study did not explore the effect of other intriguing variables, such as emotion regulation ability, social function, and personality traits. Third, as man and woman students may have different web-based behaviors, future studies can include gender as an independent variable to compare the differences between genders in the IA symptom network. Several points of strength should also be considered. To the best of our knowledge, so far, few studies have used the network analysis model and IAT scale to analyze IA. Traditional game addiction interventions often lack targeted goals. This study suggests that there are core symptoms of game addiction that are closely related to other symptoms. Intervention for these core symptoms may lead to better efficacy. Previous studies have suggested this, and a network analysis of the Sequenced Treatment Alternatives to Relieve Depression study has revealed the importance of *sad mood* and *anhedonia* in nonpsychotic depressive disorder [39]. However, further studies are needed to confirm this finding. In addition, we analyzed the network differences between the IA samples and NIA samples to make the study’s conclusions more reliable for the development of new intervention measures.

### Conclusions

In conclusion, the network is different between IA and NIA. The influence on daily life and learning is the outcome symptom of IA. Craving, losing control, and boredom are key symptoms that activate other symptoms of IA. Withholding web-based information is a key symptom of IA. The anonymity of the internet is a potential factor affecting IA. The DSM-5 diagnostic criteria for IA are associated with key symptoms and have low correlation with outcome symptoms. Determining the core symptoms of IA and the link between IAT and DSM-5 diagnostic criteria are helpful in improving the pertinence and effectiveness of IA treatment.

## Abbreviations

CS: central stability
DSM-5: Diagnostic and Statistical Manual of Mental Disorders–5th edition
IA: internet addiction
IAT: Internet Addiction Test
ICD-11: International Classification of Diseases–11th revision
IGDS9-SF: 9-item Internet Gaming Disorder Scale–Short Form
NIA: non–internet addiction

## Data Availability

The analysis code and original data are available at the Open Science Framework [40].
